# *In-situ* DRIFT investigation of photocatalytic reduction and oxidation properties of SiO_2_@α-Fe_2_O_3_ core-shell decorated RGO nanocomposite

**DOI:** 10.1038/s41598-020-59037-9

**Published:** 2020-02-07

**Authors:** Uma Kasimayan, Arjun Nadarajan, Chandra Mohan Singaravelu, Guan-Ting Pan, Jothivenkatachalam Kandasamy, Thomas C.-K. Yang, Ja-Hon Lin

**Affiliations:** 1 Department of Electro-Optical Engineering, National Taipei University of Technology, Taipei, Taiwan 106; 2 Department of Chemical Engineering and Biotechnology, National Taipei University of Technology, Taipei, Taiwan 106; 3 Department of Chemistry, Anna University, BIT Campus, Tiruchirappalli, 620024 Tamil Nadu, India

**Keywords:** Chemical engineering, Photocatalysis

## Abstract

In this work, SiO_2_@α-Fe_2_O_3_ core-shell decorated RGO nanocomposites were prepared via a simple sol-gel method. The nanocomposites were prepared with different weight percentages (10, 30, and 50 wt %) of the SiO_2_@α-Fe_2_O_3_ core-shell on RGO, and the effects on the structural and optical properties were identified. The photocatalytic reduction and oxidation properties of the nanocomposites in the gas phase were assessed through the reduction of CO_2_ and oxidation of ethanol using *in-situ* diffuse-reflectance infrared fourier transform spectroscopy (DRIFT). The prepared nanocomposite with (30 wt %) of SiO_2_@α-Fe_2_O_3_ showed superior photocatalytic activity for the gas phase reduction of CO_2_ and oxidation of ethanol. Enhancement in the activity was also perceived when the light irradiation was coupled with thermal treatment. The DRIFT results for the nanocomposites indicate the active chemical conversion kinetics of the redox catalytic effect in the reduction of CO_2_ and oxidation of ethanol. Further, the evaluation of photoelectrochemical CO_2_ reduction performance of nanocomposites was acquired by linear sweep voltammetry (LSV), and the results showed a significant improvement in the onset-potential (–0.58 V) for the RGO (30 wt %)-SiO_2_@α-Fe_2_O_3_ nanocomposite.

## Introduction

Photocatalysis is an extensively studied process by many researchers to deal with various applications mostly on energy and environmental problems. In recent years, researchers have been focused on reduction of CO_2_ into fuels such as carbon monoxide, hydrocarbons, and alcohol using solar driven photocatalysts^1,2^. Under the irradiation of light on the photocatalyst, the electrons in the CB and holes in the VB could be involved simultaneously in the reduction and oxidation reactions, respectively. Thermodynamically, the reduction of CO_2_ is an uphill reaction, so the band edge position of CB should be more negative than the reduction potential of CO_2_^3,4^. Meanwhile, for water oxidation, the VB band edge should be more positive. The efficiency of CO_2_ reduction is determined by the various kinetic parameters, which is mainly from binding of CO_2_ with the catalyst surface. This process takes place with reaction of CO_2_ + 2^e−^ → CO + ^1^/_2_O_2_, which combined with free energy of 257 KJ/mol. Finally, the CO would react with hydrogen to form formate and carbonates. Besides, controlled oxidation of ethanol is very demanding for the application of bioethanol, which is preferred for cost-effective organic synthesis^5,6^. Ethanol could be oxidized under light irradiation using photocatalyst materials. Photo-induced electrons and holes from the catalyst could react with O_2_ and H_2_O, which generates the reactive oxygen species and.OH, and these species oxidize the adsorbed ethanol.

There are many methods which can be used for the reduction of CO_2_. Electrochemical^7^, photo-catalytic^8^, and hydrothermal^9,10^ methods have all been used, but it is the electrochemical and photocatalytic conversion of CO_2_ into fuels such as CO, hydrocarbons, and alcohol which has attracted the most attention^11^. The photooxidation process is essential for the production of organic materials such as acetaldehyde^6^, butadiene^12^, acetone^5^ and other compounds. There are various materials such as Au-TiO_2_, V_2_O_5_-TiO_2_^13,14^ and Ag and Au deposited on Ceria^15^ utilized for oxidation with low temperatures which maintain the selectively of the catalyst towards the desired products of CO, CO_2_ and formate species.

Various kinds of metal oxide semiconductors have been investigated for CO_2_ photoreduction, including TiO_2_, ZnO and α-Fe_2_O_3_^16–18^. Among the various nanomaterials, the focus has been on α-Fe_2_O_3_ because of its stability and abundance^19,20^. It has been shown that the n-type α-Fe_2_O_3_ semiconductor offers better light visible absorption, a large surface area, unique surface properties, strong stability and cost-effectiveness^21–23^. These excellent properties should lead to improvement in the low potential efficiency of the wide bandgap semiconductors. For further improvement in catalytic efficiency, it is necessary to tune the crystalline structure and the surface properties of α-Fe_2_O_3_^21^. Moreover, to improve the photocatalytic reactivity of α-Fe_2_O_3_, it can be fabricated with various materials such as SnO_2_, TiO_2_, Pd, and graphene. The α-Fe_2_O_3_ nanomaterials can be synthesized by mechanochemical processing^24^, spray precipitation^25^, hydrothermal processing^26,27^ and sol-gel methods^28,29^. Of these, the sol-gel method is not only a very effective, non-toxic, low-cost method, but it is also easily adaptable for the preparation of hematite nanoparticles. This method is also the most attractive for the preparation of 1D nanostructured materials for improved solar energy conversion. It has been found that small changes in the concentration of the solvents produce changes in the size and morphology of particles with large surface area and good crystallinity.

Carbon is one of the more readily available elements, and has been used in various applications including batteries^30^, supercapacitors^31^, fuel cells^32^ and photocatalysts^33^. Graphene has also recently emerged as a material of interest because of its two-dimensional structure, superior optical and electrical properties, and the fact that photoactive materials can be immobilized on it. Among these carbon materials, graphene oxide and reduced graphene oxide are very effective for controlling the photocatalytic reaction^34^. The bandgap of GO can be triggered with its degree of reduction. The fully oxidized GO and partially oxidized GO can perform as an insulator and semiconductor, respectively. The presence of oxygen on the carbon plane triggers the RGO to exhibit as a p-type semiconductor. It has been shown that the coupling of graphene with a semiconductor improves its photocatalytic performance. When RGO is combined with a metal oxide, it acts as a supporting matrix because of its stability and conductivity^35^. Also, these supportive RGO nanocomposites can maintain their structural integrity, which in turn enhances the electrochemical reduction and makes them suitable for other applications^35,36^.

DRIFT is an estabilished process which can be applied to measure the conversion of gaseous products into the various useful components at low temperatures^37^. The previous reports revealed that the photocatalytic reduction of CO_2_ was enhanced using selective behavior of a catalyst that helped to reduce the CO_2_ to methanol^38^. Besides, notably, the information about the reaction with oxygen in semiconductor materials emerged^13,39,40^. Interestingly, the V_2_O_5_-TiO_2_ semiconductor was used to study the ethanol-oxidation by DRIFT, showing the enhanced photocatalytic activity under visible light^13^.

In this present investigation, the SiO_2_@α-Fe_2_O_3_ core-shell decorated RGO nanocomposites were prepared by the simple sol-gel method. We hypothesized the synergistic effect of the SiO_2_@α-Fe_2_O_3_ core-shell on the RGO which would enhance the gas phase reduction and oxidation behavior of the catalyst. The DRIFT study reveals the adsorption of CO_2_ species on the surfaces, and facilitates the reduction of CO_2_ into the various products. Besides, it also works on the oxidation of ethanol into final products of CO_2_ with intermediates, implying the suppression of the electron-hole recombination rate and improving the catalytic activity. Photoelectrochemical performance on reduction of CO_2_ was also investigated, and strongly indicated the improvement in the catalytic behavior. These studies indicate that this heterojunction photocatalyst of the SiO_2_@α-Fe_2_O_3_ core-shell decorated RGO nanocomposites might be a unique system for both reduction and oxidation reactions.

## Experiments

The graphene oxide (GO) precursor material was prepared by the well-known Modified Hummer’s methods as reported previously^41,42^. First, we prepared the SiO_2_ material as described in our previous report^43^. Nanosphere SiO_2_ was prepared using tetraethyl orthosilicate (TEOS), anhydrous ethanol, and water. This solution was stirred for 30 min. We then added the ammonia solution, stirring for 3 min, followed by the addition of anhydrous ethanol with continuous stirring for 12 h. Finally, the obtained precipitate was washed with ethanol and water, then dried in an oven for 5 h at 90 °C. Furthermore, to obtain a hematite product with small nanoscaled particles, we used an iron (III) nitrate monohydrate precursor. In this case, the pH of the solution was maintained at 11.5 by the addition of a sodium hydroxide aqueous solution. Then 50 mg of dispered solution of SiO_2_ nanosphere was added with an iron nitrate monohydrate precursor. This mixer was stirred for 12 h at 95 °C, and the FeOOH-coated SiO_2_ nanospheres were separated by centrifugation. The resulting material SiO_2_@α-Fe_2_O_3_ was dried in a vacuum oven at 60 °C for 6 h and finally annealed at 450 °C for 3 h. The RGO-SiO_2_@α-Fe_2_O_3_ was prepared by dispersing a mixture of graphene oxide and SiO_2_@α-Fe_2_O_3_ (10, 30, and 50%) nanoparticles in water with effective stirring for 1 h with the addition of 600 μl of hydrazine hydrate. The mixture was then refluxed at 75 °C for 6 h under stable conditions and then dried.

### DRIFT measurement

The DRIFT performance was analyzed with an FT-IR Perkin Elmer using an MCT detector. The CaF_2_ window was used for the diffuse reflectance measurement with 32 scans for each spectrum with a resolution of 4 cm^−1^. The specimen chamber loaded with the sample was purged with flowing nitrogen for 1 h to remove air. The CO_2_/H_2_O vapor mixture was continuously poured into a closed condition of the entire DRIFT cell chamber for 30 min. Subsequently, the UV-visible (350–750 nm) light was irradiated on the catalyst through the third window of the DRIFT cell, and the spectra were measured at different times and temperatures. During irradiation, the infrared absorbance within the wavenumber range of 4000–1000 cm^−1^ was recorded at certain time points. For the photo-oxidation of the ethanol process, the DRIFT cell was occupied with 20 mg of sample. The pure ethanol (99%) combined with oxygen gas was purged into the DRIFT chamber at a flow rate of 30 cc/min. Then the UV-visible light was continuously illuminated on the DRIFT cell, and the spectrum was observed at regular intervals of 3 min.

### Electrochemical measurement

The electrochemical impedance measurement was carried out using a Zive Potentiostate, SP100. Electrochemical impedance measurement (EIS) was used to observe the interfacial properties of the RGO, and the RGO-coated SiO_2_@α-Fe_2_O_3_ nanocomposites with various ratios of SiO_2_@α-Fe_2_O_3_ core-shell with modified GCE in 0.1 M KCl containing 5 mM of Fe(CN)_6_^3−/4−^, wherein bias potential = 0 V, amplitude = 5 mV, and Frequency = 0.1Hz–100 kHz were applied. Linear sweep voltammetry was carried out at room temperature with a three-electrode system. The ITO glass functioned as the working electrode, with Ag/AgCl (saturated 3 M KCl) as the reference electrode and the counter electrode as the platinum. The working electrode was prepared by mixing RGO-SiO_2_@α-Fe_2_O_3_ with 2 ml of distilled water followed by sonication for 45 min. Then the mixture was deposited on ITO and dried at 50 °C for 2 h. The RGO-SiO_2_@α-Fe_2_O_3_ modified electrode was then immersed in a 0.1 M KHCO_3_ electrolyte solution with a pH of 8.4. The latter was purified by pre-electrolysis under N_2_ gas. Prior to the CO_2_ reduction process, CO_2_ gas (99.999%) was bubbled to saturate the KHCO_3_ electrode solution for 1 hr. During the experiment, CO_2_ was bubbled into the electrolyte at a flow rate of 10 sccm. The electrolyte was stirred using a Teflon-coated magnetic stirrer bar at 1500 rotations per minute (rpm) to enhance the mass flow of CO_2_ to the working electrode. Then the LSV was measured between 0 and −1.4 eV vs. Ag/AgCl at 50 mVs^−1^ under UV-vis (350 W mercury-xenon lamp) light irradiation^44^. The measured values were calculated for RHE from the following equation:

1 $${{\rm{E}}}_{{\rm{RHE}}}\,({\rm{V}})={{\rm{E}}}_{{\rm{Ag}}/{\rm{AgCl}}}\,({\rm{V}})+0.197\,{\rm{V}}+(0.059\,{\rm{V}}\times {\rm{pH}}).$$

### Characterization

The crystallographic structure of the samples was measured by XRD (Analytical X’Pert 246 PRO) using filtered CuKα radiation (λ = 1.5418 Å). The morphologies of the nanocomposites were studied with field emission scanning electron microscopy (FE-SEM) with a JEOL JSM-7100F. Transmission electron microscopy (TEM) was analyzed by JEOL JEM2100F. The elemental composition was observed from the X-ray photoelectron spectra (XPS) obtained using a JEOL, JPS-9030.

## Results and Discussion

### Material characterization

**Figure 1:**
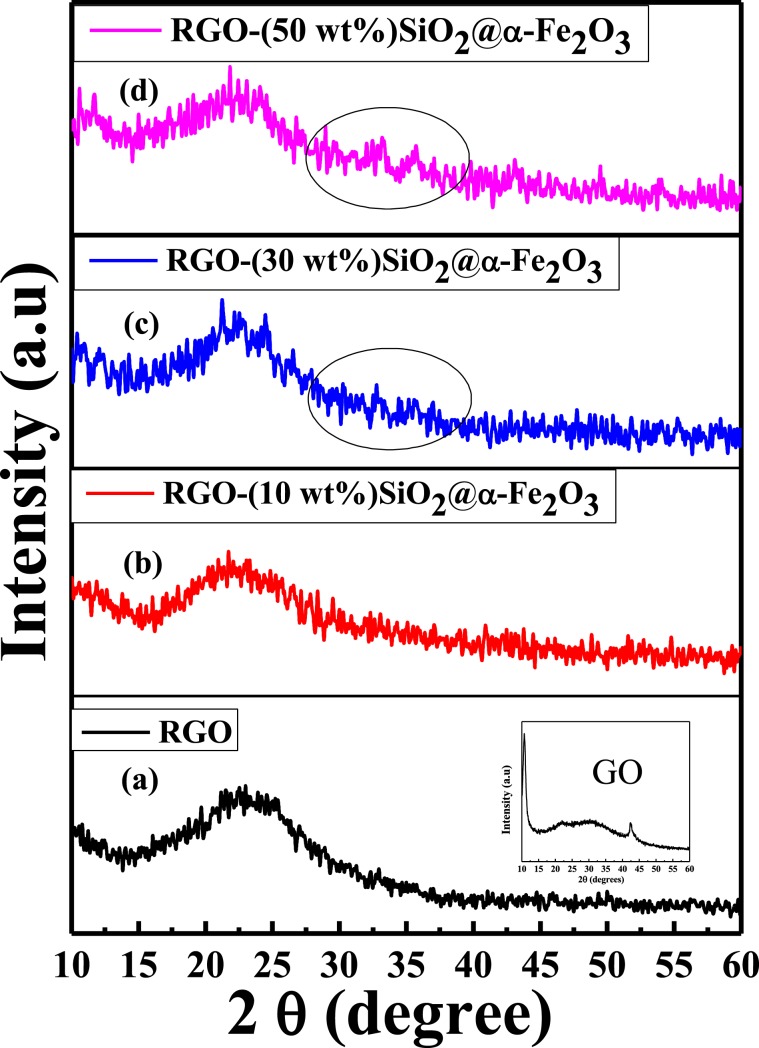
XRD patterns of RGO and RGO-SiO_2_@α-Fe_2_O_3_ core-shell nanocomposites with different weight percentages of SiO_2_@α-Fe_2_O_3_.

The XRD profile of the as-prepared samples was recorded to confirm the crystalline behavior of the GO, RGO and RGO-SiO_2_@α-Fe_2_O_3_ (10, 30 and 50 wt %) nanocomposites, and is presented in Fig. 1. The observed sharp peak at 12^o^, from the inset Fig. 1(a) confirms the crystalline GO as prepared by Hummer’s method. The broad peak 2θ at 24^o^ shows the reduced form of GO after refluxing, as shown in Fig. 1. The observed RGO diffraction peaks can be attributed to the 002 plane due to the partial removal of oxygen functionalities from the stacked Sp_2_ layers^45^. In the case of the nanocomposites (Fig. 1(b)–(d)), a similar broad 2θ peak at 24° was also observed, indicating the formation of SiO_2_ and RGO merged together. Moreover, the two small 2θ peaks at 32.2° and 34° were also observed with respect to the weight percentage of the SiO_2_@α-Fe_2_O_3_ core-shell nanocomposites. The observed 2θ peaks at 32.2° and 34° can be assigned to the (104) and (110) planes of the α-Fe_2_O_3_ hematite particles (JCPDS 33–0664). However, these two corresponding peaks for the α-Fe_2_O_3_ hematite particles were not observable in (10 wt %) of SiO_2_@α-Fe_2_O_3_ core-shell in the RGO nanocomposites due to its low concentration. By increasing the concentration to 30 and 50 wt %, diffraction peaks with low intensity were observed for the α-Fe_2_O_3_, indicating the presence of SiO_2_@α-Fe_2_O_3_ core-shell nanocomposites on the RGO nanosheets.

**Figure 2:**
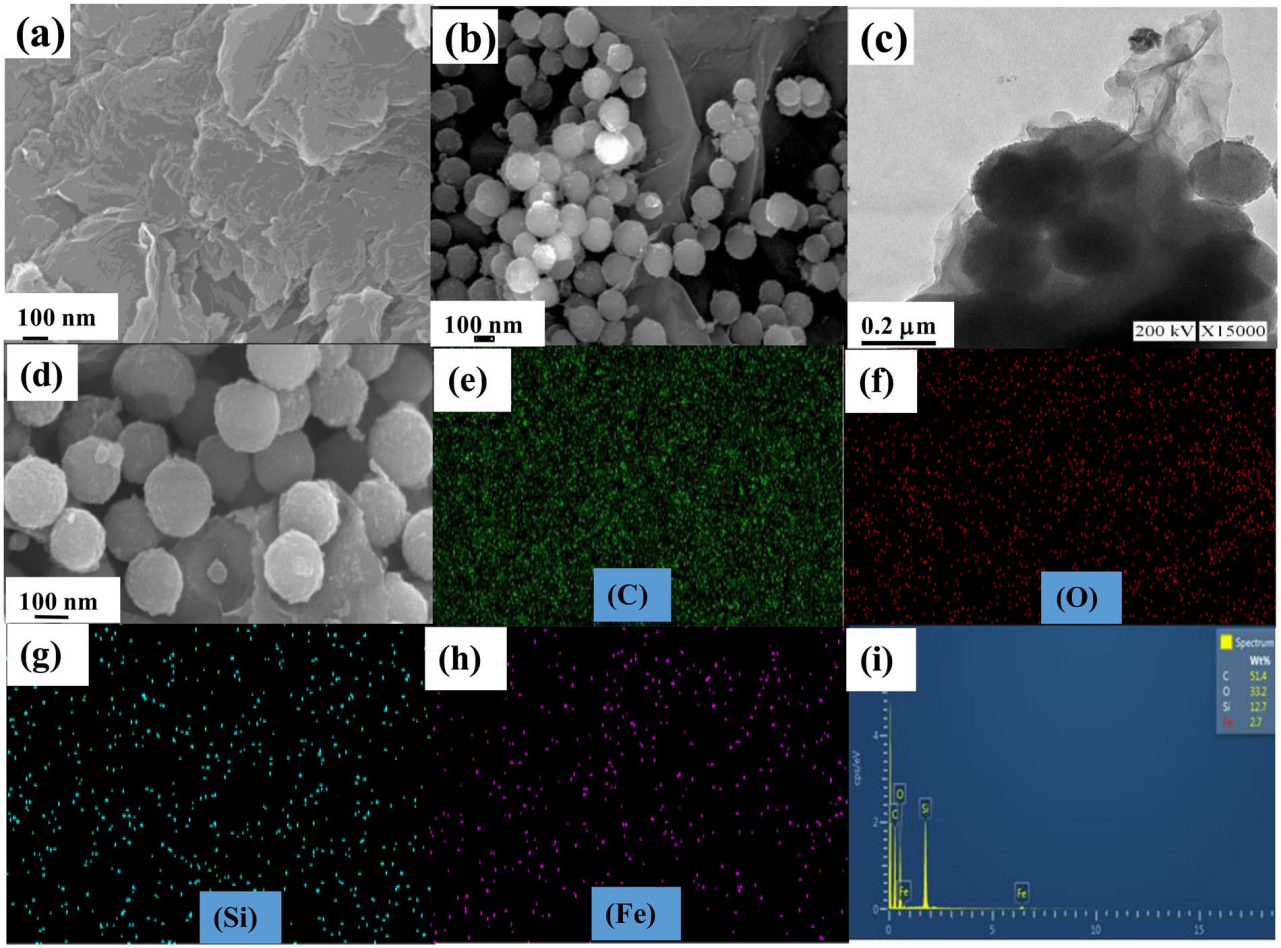
FE-SEM image of (**a**) RGO, (**b**) RGO-(30 wt%) SiO_2_@α-Fe_2_O_3_ core-shell nanocomposites and (**c**) TEM picture of RGO-(30 wt%) SiO_2_@α-Fe_2_O_3_ core-shell nanocomposites and (**d**–**i**) FE-SEM image and their corresponding elemental mapping and EDX.

The surface morphology of the prepared nanocomposites was analyzed using the FE-SEM and TEM techniques; the images are given in Fig. 2. The FE-SEM image (Fig. 2a) for the RGO shows transparent layers of RGO with many wrinkled sheets. Figure 2(b) shows the FE-SEM image of the RGO-(30 wt %) SiO_2_@α-Fe_2_O_3_ nanocomposites, and depicts the distribution of the SiO_2_@α-Fe_2_O_3_ core-shell decorated RGO sheets. The TEM analysis of the SiO_2_ and SiO_2_@α-Fe_2_O_3_ nanospheres obviously confirms the formation of the core-shell structured SiO_2_@α-Fe_2_O_3_ as shown in S1(f), (g) and (h). Figure 2(c) shows a TEM image of the (30 wt %)-SiO_2_@α-Fe_2_O_3_ nanocomposites on the RGO nanosheets. This confirms the existence of SiO_2_@α-Fe_2_O_3_ core-shell nanocomposites and the thorough distribution of the reduced graphene oxide sheets. Even after the reduction of GO, the α-Fe_2_O_3_ nanoparticles are still present on the SiO_2_ nanospheres, as shown in Fig. 2(c). The mapping results confirm that each element is distributed within the nanocomposites as shown in Fig. 2(d). The corresponding EDX analysis confirms the presence of elements such as Si, Fe, O and C in the nanocomposites.

**Figure 3:**
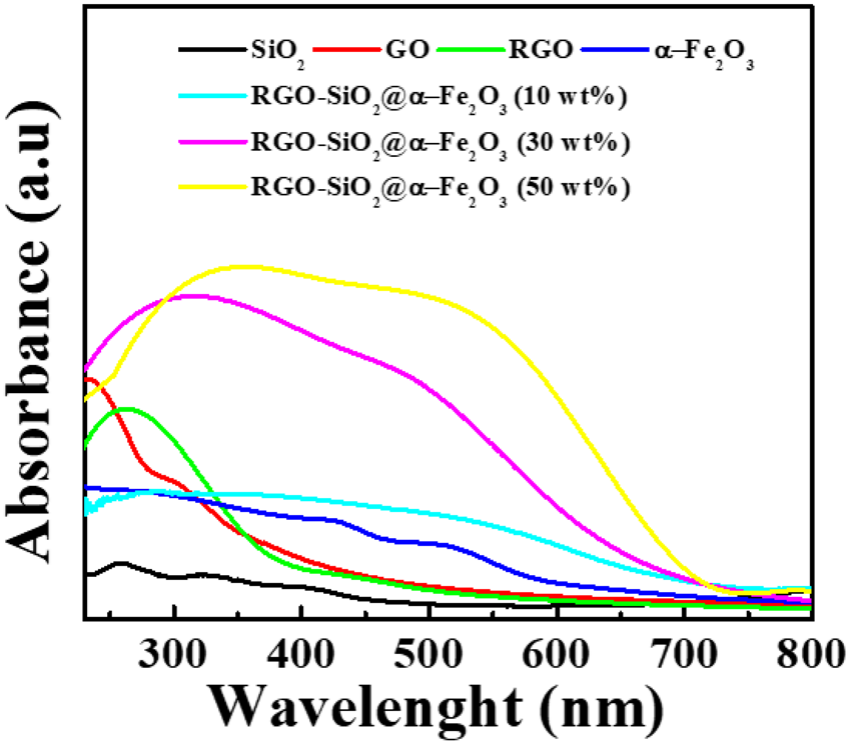
UV-vis (DRS) spectra of SiO_2_, RGO, α-Fe_2_O_3,_ SiO_2_@α-Fe_2_O_3,_ and RGO-SiO_2_@α-Fe_2_O_3_ core-shell nanocomposites with different weight percentages of SiO_2_@α-Fe_2_O_3_.

UV-vis diffuse reflectance spectra were recorded to examine the optical properties of the prepared nanocomposites, and are presented in Fig. 3. The absorption spectrum for SiO_2_ nanospheres shows band at the UV region, however, after the formation of core shell with hematite α-Fe_2_O_3_ shows broad band in the visible region. Correspondingly, the absorption band for the RGO- SiO_2_@α-Fe_2_O_3_ nanocomposites shifted towards more visible region with respect to the weight percentage of SiO_2_@α-Fe_2_O_3_. This clearly indicates that the prepared nanocomposites are visibly active for the photocatalytic applications. The band gap of RGO and α-Fe_2_O_3_ were calculated by extrapolating the rising segment of the UV spectrum at zero absorption with the support of the Tauc plot as shown in S2. The band gap for the hematite α-Fe_2_O_3_ was found to be 2.2 eV. Besides, the band gap for bare RGO was calculated from the band edge of spectra through Tauc plot and found to be 3.6 eV which is comparable with the reported value^46^. Mostly, the bandgap for the RGO was valued to be in the range of 1.9 to 3.6 eV, which can be easily tuned as a function of reduction^47^. In the case of RGO-SiO_2_@α-Fe_2_O_3_ nanocomposites, *in-situ* chemical reduction of GO was followed. The accumulation of SiO_2_@α-Fe_2_O_3_ core-shell on the graphene oxide layer initiates the formation of reduced form of GO (RGO). After the decoration of SiO_2_@α-Fe_2_O_3_ core-shell on the graphene oxide layer, due to the degree of reduction the band edge corresponding to the RGO prolong towards the visible region. So, the obtained bandgap for RGO after the reduction by the SiO_2_@α-Fe_2_O_3_ core shell was found to be 2.1 eV, which is shown in S2. These observed changes in the band gap plays significant role in the oxidation and reduction properties of prepared nanocomposites under the visible light irradiation.

**Figure 4:**
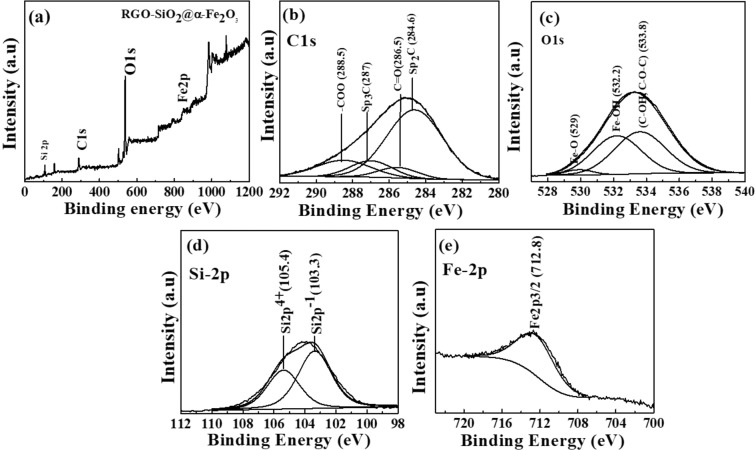
(**a**) XPS survey, (**b**) C1s, (c) O1s, (**d**) Si-2p and (**e**) Fe-2p spectra of RGO- (30 wt%) SiO_2_@α-Fe_2_O_3_ core-shell nanocomposites.

Figure 4(a) shows the XPS survey spectra of the RGO-SiO_2_@α-Fe_2_O_3_ nanocomposites. High resolution XPS spectra were also recorded to determine the chemical structure and oxidation states of elements present in the RGO-SiO_2_@α-Fe_2_O_3_ nanocomposite. The peaks observed at 284.6, 287 and 288.5 eV correspond to Sp_2_C, Sp_3_C, and –COO, respectively. The peak located at 286.5 eV is associated with the C = O groups. This proves the formation of reduced graphene oxide after the reduction of GO as shown in Fig. 4(b). The typical peaks from Fig. 4(c) at 532.2 and 533.4 could be allocated to O 1 s, indicating O in the O^2−^ state^48^. The peak at 529 eV corresponds to the external Fe-O group adsorbed on the surface. The binding energies of 103.3 and 105.4 eV are attributed to Si 2p^−1^ and Si 2p^4+^, respectively, in SiO_2_ as shown in Fig. 4(d)^49^. Figure 4(e) displays the XPS of Fe 2p with the binding energies at 712.8 assigned to the Fe 2p_3/2_,^50,51^. The C1s spectra of RGO and RGO-SiO_2_@α-Fe_2_O_3_ nanocomposites are shown in S3. The C1s spectrum reveals a dominant C–C (284.6 eV) peak present in the RGO and RGO-SiO_2_@α-Fe_2_O_3_ nanocomposites as shown in S3 (a) and (b). Further, the reduction in peak intensity is observed for epoxy C–O at 286.5 eV, carbonyl C = O at 287 eV, and carboxyl O = C–O at 288.5 eV, which indicates the reduction of oxygen-containing functional groups in the RGO-SiO_2_@α-Fe_2_O_3_ nanocomposites when compared with RGO. The O1s spectra of RGO showed three peaks at 530, 532.3, and 533.4 eV (S3 (c). The binding energy at 530 eV is attributed to doubly bonded oxygen atoms and the peak at 532.3 eV is linked to singly bonded oxygen-containing functional groups. Further, the presence of a peak at 533.4 eV confirms the bonded oxygen atoms in carboxyl and ester functionalities. In the α-Fe_2_O_3_ spectra, the peak at 529 eV is attributed to Fe–O which confirms the interaction between Fe and oxygen, and the peak at 532.2 eV is ascribed to the C–OH groups S3 (d). The O 1 s spectrum of RGO-SiO_2_@α-Fe_2_O_3_ possesses similar peaks as in the O1s spectrum of RGO as seen in S3 (e). The slight reduction in a number of oxygen-containing groups in O1s (C–OH, C–O–C) demonstrates the effective reduction of oxygen. Further, the broad emerging peak at 532.2 eV corresponds to C–O–Fe, suggesting the linkage formed between α-Fe_2_O_3_ and RGO. The peak at 532.2 eV demonstrates the existence of Fe–O in the hybrid that is beneficial to the adsorption of heavy ions. The peak at 712.0 eV is assigned to Fe2p_3/2_ of α-Fe_2_O_3_ as seen in Fig. S3(f) and it is shifted to lower binding energy in SiO_2_@α-Fe_2_O_3_ and RGO-SiO_2_@α-Fe_2_O_3_ composites, which confirms the existence of iron oxides. This indicates that the electron transfer process is taking place between RGO and α-Fe_2_O_3_ that is favorable for increasing the conductivity. The binding energies of 103.6 are attributed to Si 2p^−1^ in SiO_2_ and are reduced after depositing α-Fe_2_O_3_ as shown in Fig. S3(g). These indicate the presence of α-Fe_2_O_3_ nanoparticles on the SiO_2_.

### Photocatalytic *In-situ* DRIFT study in the gas phase

#### Photocatalytic reduction of CO_2_

**Figure 5:**
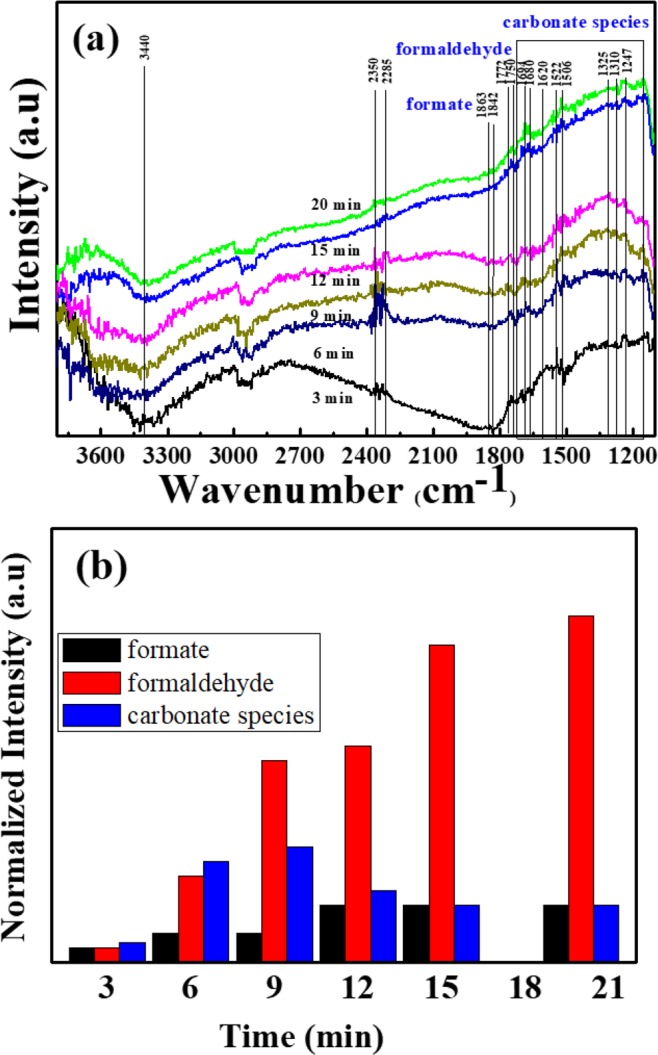
(**a**) DRIFT-IR spectra of photocatalytic CO_2_ reduction in a flow of CO_2_/H_2_O vapor mixture as a function of different irradiation time. (**b**) Evaluation of formate, formaldehyde and carbonate species as the function of the different time interval.

Photocatalytic reduction of CO_2_ was evaluated using the *In-situ* DRIFT cell under UV-vis light to study the gas-phase reduction of CO_2_ and their transformation products on the catalyst surface. Figure 5(a) shows the absorbance spectra as a function of irradiation time using RGO-(30 wt%) SiO_2_@α-Fe_2_O_3_ nanocomposites. Broadband was observed at 3440 cm^−1^, which is attributed to the OH stretching. This indicates that the surface hydroxyl group was generated, which is observed from the dissociative adsorption of H_2_O on the RGO-(30 wt%) SiO_2_@α-Fe_2_O_3_ core-shell nanocomposites. The peaks at 2285 and 2350 cm^−1^ confirm the presence of adsorbed CO_2_^−^ on the surface of RGO-(30 wt %) SiO_2_@α-Fe_2_O_3_ nanocomposites. Initially, the CO_2_^−^ is the essential group for photoreduction of CO_2_, confirming the interaction between molecular carbon dioxide and carbonate ions on the surface of the catalyst. It is interesting to note that these CO_2_^−^ species are consequently adsorbed from a flow of CO_2_/H_2_O vapor mixture which is crucial for photoreduction. The photocatalytic conversion of CO_2_ into CH_4_ and CH_3_OH is a multistep process with an upward reaction, and it causes a highly positive change in Gibbs free energy. Consistently, the multiple peaks corresponding to the intermediates such as adsorbed carbonates, adsorbed formate (HCOO), and molecularly adsorbed formaldehyde (HCHO) were observed at room temperature under irradiation. The assigned asymmetric CO stretching and symmetric CO stretching carbonate bands were observed at 1680, 1620, 1522, 1506 and 1310 cm^−1^. The peaks observed at 1863, 1842, 1694 and 1325 cm^−1^ are assigned to adsorbed HCOO species on the surface of the nanocomposites. The peaks corresponding to the HCHO are found at 1772, 1710 and 1247 cm^−1^ and these results agree well with the previous report^38^. The observed carbonates are primary intermediate products formed during CO_2_ conversion due to the proton transfer when increasing the light irradiation from 3 to 20 min on the RGO-(30 wt %) SiO_2_@α-Fe_2_O_3_ nanocomposites. It is worth noting that the carbonate species may react with multiple electrons and hydrogen and then convert to formate species^52,53^. This is confirmed with decreasing carbonate peaks with increasing formate and formaldehyde peaks when increasing light irradiation from 15 to 20 min. Under the irradiation of light, the photogenerated electron-hole pairs were formed and led to the charge separation state. Due to the core-shell formation of SiO_2_ with α-Fe_2_O_3_ nanoparticles, the charge recombination was suppressed. The photogenerated holes react with the adsorbed water molecules and generate H^+^ ions. These H^+^ ions and photogenerated electrons transferred through the RGO and initiated reduction with the adsorbed CO_2,_ leading to consecutive catalytic reactions. Unfortunately, the characteristic peaks for other intermediates such as CH_3_O and CH_4_ are not observed, which might be due to the partial reduction of CO_2_ and the low affinity on the sample surface. Figure 5(b) shows the normalized intensity as a function of the different time intervals at room temperature. This indicates that the formate and formaldehyde increase with the increase in time from 3 to 15 min. However, the carbonate species decreased after irradiating the UV-vis light from 11 to 15 min. These results confirm that nanocomposites show an effective CO_2_ reduction property at room temperature, and it is not possible to trace the generation of the intermediates.

**Figure 6:**
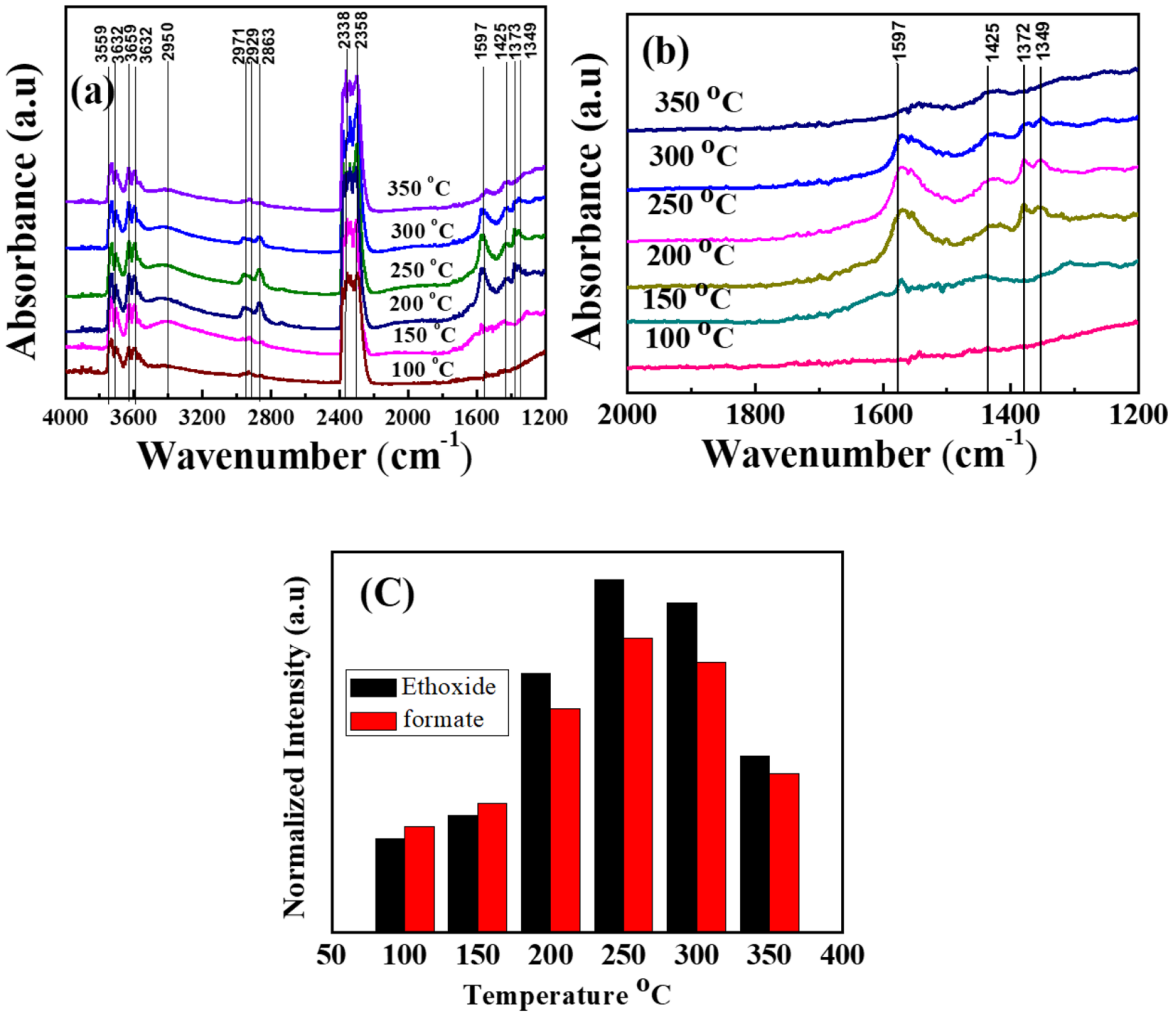
(**a**) DRIFT-IR spectra of RGO- (30 wt%) SiO_2_@α-Fe_2_O_3_ core-shell nanocomposites from 100 to 350 °C. (**b**) Magnified images of Fig. 6(a) (1200–1000 cm^−1^). (**c**) Ethanol and formate species from the RGO- (30 wt%) SiO_2_@α-Fe_2_O_3_ core-shell nanocomposites with various temperatures.

Further, in order to improve the CO_2_ reduction and explore their photocatalytic conversion process, we applied different temperatures as the photothermal effect on the RGO (30 wt %) SiO_2_@ α-Fe_2_O_3_ nanocomposites as shown in Fig. 6(a). When introducing the thermal effect on the catalyst surface, the new peaks were observed in the region between 1700 and 1000 cm^−1^ which are attributed to the bicarbonate and adsorbed formate [64, 65]. The magnification of the region between 1700 and 1000 cm^−1^ is shown in Fig. 6(b). The observed band at 1425 cm^−1^ is assigned to bicarbonate, and the remaining peaks at 1349,1372 and 1597 cm^−1^ are allocated to the adsorbed formate. At the temperatures of 100 and 150 °C, low-intensity bands of adsorbed formate were observed. These bands were much stronger, were detected at 250 °C and stayed longer up to 300 °C. When the temperature increased up to 300 °C, the bicarbonate band did not show a significant difference in peak intensity; this confirms that carbonate could generate ethoxy intermediates. Also, as the temperature increased from 100 to 350 °C, the absorption peaks at 2338 and 2357 cm^−1^ corresponding to the CO_2_ gas phase indicate the strong bonding interaction between CO_2_ and the carbon ions^54^. Moreover, these bands are ascribed to the coordination of surface Lewis acid sites of CO_2_ molecules. Also, the bands at 3759, 3732, 3632 and 3659 cm^−1^ reflect the –OH stretching vibration of weak H bonding from the H_2_O vapor. Moreover, the bands which appear between the 2800–2400 cm^−1^ regions are probably due to the ethoxide species of –CH stretching modes at 2971, 2929, and 2863 cm^−1^ (CH_3_(as), CH_2_(as) and CH_3_(s)^55,56^. These -CH species suggest the generation of the various hydrocarbonates, and more of the formate species were produced on the surface of the nanocomposites catalyst at temperatures between 150 and 350 °C. These results indicate that the strongest chemical adsorption and activation takes place between 150 to 300 °C under UV-vis light. Furthermore, physical-desorption of CO_2_ reduction occurred at low temperatures from the carbonate species and converted to the ethanol product from chemi-desorption at high temperatures^57,58^.

Figure 6(c) shows the ethoxide and formate species as the function of varying temperature from 100 to 350 °C. The RGO-(30 wt %) SiO_2_@α-Fe_2_O_3_ nanocomposites show the best ethanol conversion at the temperature of 250 °C. S4 implies CO_2_ reduction under dark and light irradiation at 250 °C. As a result, the RGO-(30 wt %) SiO_2_@α-Fe_2_O_3_ core-shell nanocomposites show the best catalytic performance under the light when compared with the dark. S5 shows the gas phase CO_2_ reduction on the α-Fe_2_O_3_, SiO_2_@α-Fe_2_O_3_ and RGO-(10, 30 and 50 wt %) SiO_2_@α-Fe_2_O_3_ nanocomposites at a reaction temperature of 250 °C. When compared with 10 and 50 wt % SiO_2_@α-Fe_2_O_3_ nanocomposites, the RGO-supported (30 wt %) SiO_2_@α-Fe_2_O_3_ shows higher peaks at 2971 and 2929 cm^−1^ that are attributed to CH_x_ and are transformed into stable linear formate. At RGO-(30 wt %) SiO_2_@ α-Fe_2_O_3_, the catalytic activity decreases the formation of hydrogen and increases the formate species when compared with other weight percentages of nanocomposites.

### Photocatalytic oxidation of ethanol

**Figure 7:**
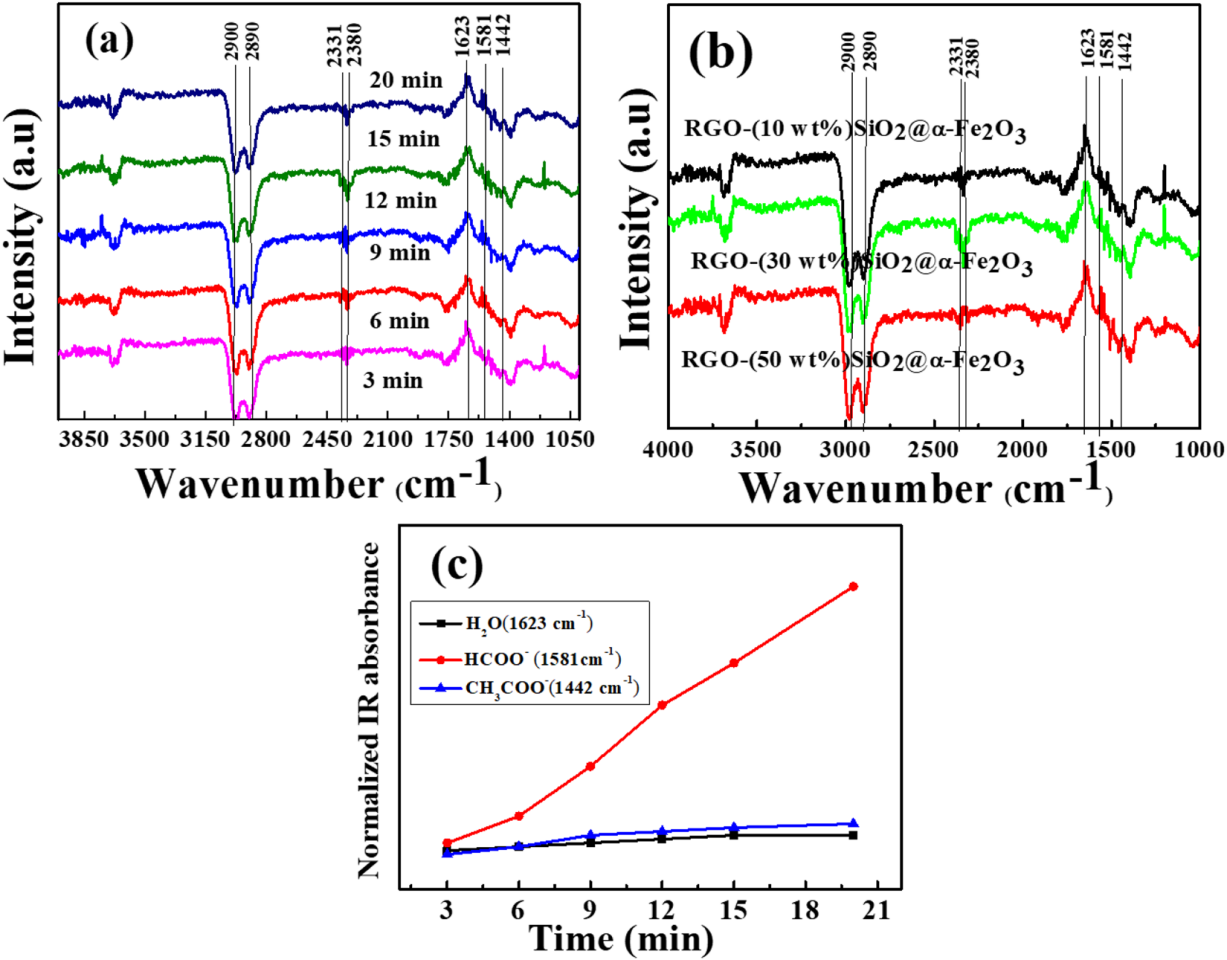
(**a**) Photooxidation of ethanol for RGO-(30 wt%)SiO_2_@α-Fe_2_O_3_ nanocomposites under Uv-vis irradiation from at different time intervals. (**b**) Photooxidation of ethanol for RGO-(10, 30 and 50 wt%) SiO_2_@α-Fe_2_O_3_ nanocomposites. (**c**) variation of normalized intensity of 1623 cm^−1^, 1581 cm^−1^ and 1442 cm^−1^ as the function of the different time interval.

In order to investigate the catalyst activity towards the reverse process of ethanol to CO_2_, the *in situ* DRIFT studies were examined to confirm the photooxidation of ethanol using RGO-SiO_2_@α-Fe_2_O_3_ nanocomposites in an oxygen atmosphere. Figure 7(a) shows the IR absorbance of RGO- (30 wt %) SiO_2_@α-Fe_2_O_3_ with different irradiation times (3, 6, 9, 12, 15 and 20 min) in a O_2_ atmosphere. The value of the IR absorbance intensity (A) was calculated from the value of the single-beam intensity (I_SB_) and the reference beam intensity (I_0_) using the following equation:

2 $${\rm{A}}=\,\log [{I}_{0}/{I}_{SB}]$$

The IR difference spectra are calculated by subtracting dark from the extension of the illumination of the UV-vis light. Before irradiation, the adsorbed ethanol peak was observed at 2890 and 2900 cm^−1^ which corresponds to the C-H vibration of ethanol. Besides, when irradiating the UV-light with different time intervals, the ethanol deformation was observed in RGO- (30 wt %) SiO_2_@α-Fe_2_O_3_ as shown in Fig. 7(a). After irradiation, the characteristic peaks of water (H_2_O), formate (HCOO^−^) and acetate (CH_3_COO^−^) were observed at 1623, 1581 and 1442 cm^−1^ respectively. Also, the band at 1581 cm^−1^ was observed as a result of the partial oxidation of ethanol, and it stayed longer when the irradiation time increased from 15 to 20 min. This is due to the oxidation of ethanol that leads to breaking down the C-C bonds in ethanol; then the holes in the valance band attack the hydrogen atom. We conclude that CH_3_COO^−^ and HCOO^−^ intermediates are accumulated during photo-oxidation, which led to the formation of CO_2_ and H_2_O as the results of ethanol oxidation^13^. During the photocatalytic oxidation reaction, the effective hot holes are formed after the irradiation of UV-vis light that might be combined with H_2_O to yield hydroxyl radicals (.OH). Further, the combination of electrons from the RGO and oxygen on the nanocomposites released O_2_^−^ radicals, which further reacted with H_2_O in order to harvest.OH radicals. Based on this continuous reaction process, the suppression of the electron-hole recombination increases the photo-oxidation reaction rate on the surface of the catalyst. Finally, the separation of electrons and holes is produced, and the hydroxyl radicals are considered to be very reactive oxidative radicals and are converted into CO_2_. The higher CO_2_ peaks were observed at 2331 and 2380 cm^−1^ for 15 and 20 min light irradiation, which confirms the high conversion ethanol oxidation rate.

Figure 7(b) shows the photooxidation of ethanol using a different concentration of SiO_2_@α-Fe_2_O_3_ in RGO with irradiation of light. From Fig. 7(b), we can determine the ethanol oxidation conversion with the detection of the acetate and aldehyde spectra and the final products of CO_2_. The oxidation of ethanol to the CO_2_ gas phase was confirmed by the presence of acetate and aldehyde spectra at 2331 and 2380 cm^−1^_,_ respectively. A high rate of ethanol oxidation was observed for RGO-(30 wt %) SiO_2_@α-Fe_2_O_3_ nanocomposites that proved the high oxygen defects^59^ and a reduction in the electron-hole recombination when compared with other nanocomposities as shown in S6. Figure 7(c) shows the normalized peak intensity of 1442, 1581, and 1623 cm^−1^ as the functions of the different time intervals. In this case, the peak at 1581 indicates the conversion of ethanol into CO_2_ when increasing the time from 3 to 20 min.

### Electrochemical analysis

**Figure 8:**
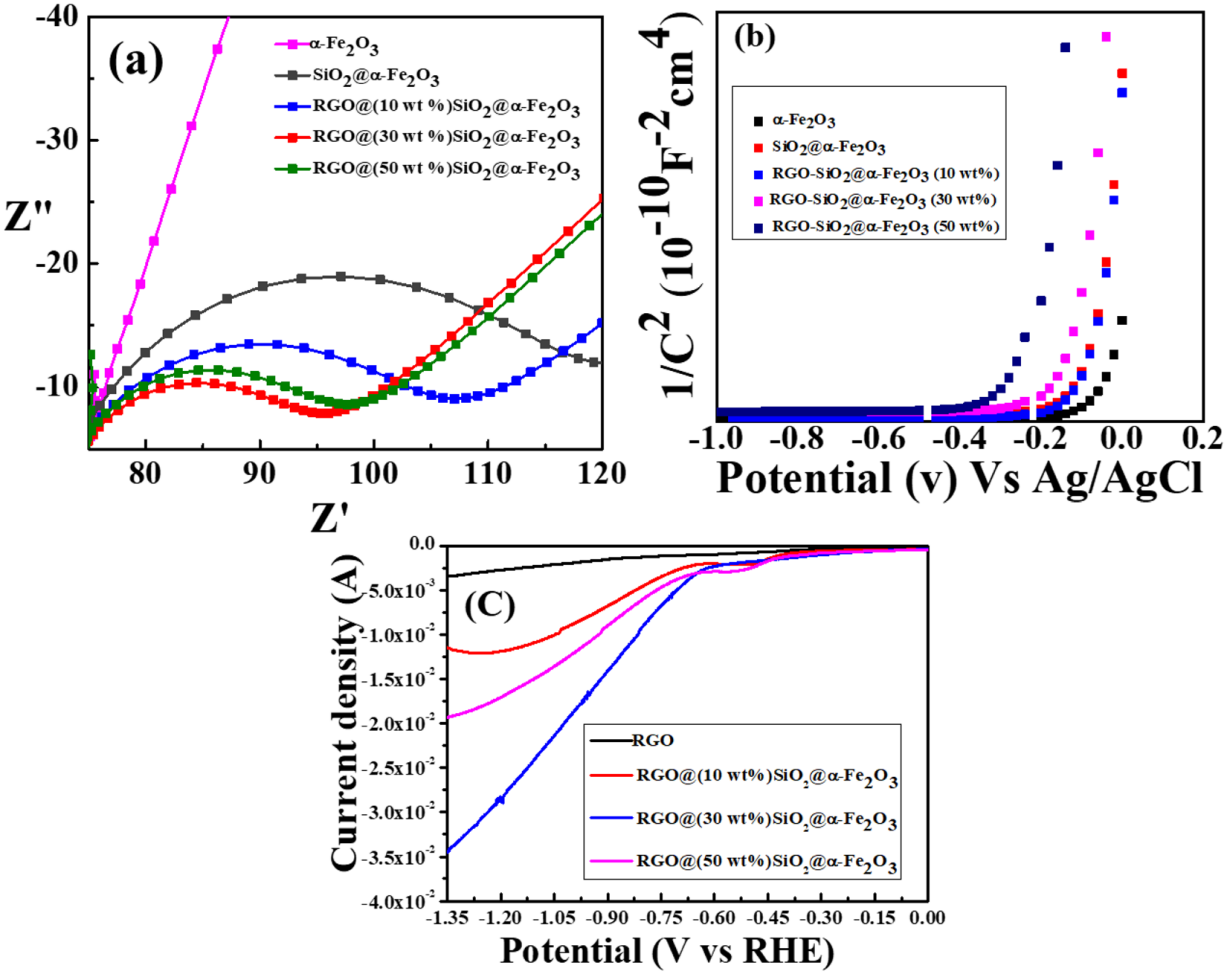
(**a**) EIS and (**b**) Mott-Schottky plot of α-Fe_2_O_3_, SiO_2_@α-Fe_2_O_3_ and RGO- SiO_2_@α-Fe_2_O_3_ core-shell nanocomposites with different weight percentages of SiO_2_@α-Fe_2_O_3_. (**c**) LSV measurement of α-Fe_2_O_3_ and RGO- SiO_2_@α-Fe_2_O_3_ core-shell nanocomposites with different weight percentage.

To analyze the electrochemical measurement, impedance was also performed for the RGO and RGO-SiO_2_@α-Fe_2_O_3_ nanocomposites. As illustrated in Fig. 8(a), the small semicircles are seen for the RGO-SiO_2_@α-Fe_2_O_3_ core-shell nanocomposites when compared to RGO due to the electron transfer impedance of the electrode surface which improves the charge transfer resistance on the catalytic surface. The diameter of the arc radius on the EIS Nyquist plot is very small in the RGO (30 wt%) - SiO_2_@α-Fe_2_O_3_ core-shell nanocomposites due to more effective separation of the photogenerated electron-hole pairs. It is also worth noting that the RGO (30 wt%) - SiO_2_@α-Fe_2_O_3_ (inset figure) has a lower resistance when compared to the SiO_2_@α-Fe_2_O_3_ and all other composites of RGO-SiO_2_@α-Fe_2_O_3_, which is indicative of the charge transfer of electrons from the SiO_2_@α-Fe_2_O_3_ core-shell to the RGO nanosheets. The smaller slope from RGO-SiO_2_@α-Fe_2_O_3_ (30 wt%) may increase in the charge carrier density and reduce in the electron-hole recombination. Figure 8(b) shows the Mott–Schottky plot of nanocomposites measured in the dark condition. These results show a positive slope in the Mott–Schottky plot which confirms an n-type semiconductor with electrons as the majority charge carriers. In order to confirm the recombination of the photogenerated electron-hole pairs, the photoluminescence (PL) spectra of α-Fe_2_O_3_, SiO_2_@α-Fe_2_O_3_ and RGO**-**SiO_2_@α-Fe_2_O_3_ nanocomposites were measured as shown in S9. The strong intense peak at 530 nm can be assigned to the recombination of photoexcited holes with electrons in α-Fe_2_O_3_^60^. However, the PL intensity of the SiO_2_@α-Fe_2_O_3_ core-shell largely decreased, indicating that the rate of recombination of electron–hole pairs has been reduced. The RGO loaded α-Fe_2_O_3_ shows minimum intensity of the PL spectra. The effective lowering of PL intensity suggests that the RGO-supported SiO_2_@α-Fe_2_O_3_ core-shell nanocomposites inhibit significant recombination of photogenerated electron-hole pairs.

### Photoelectrochemical reduction of CO_2_

Further, to confirm the CO_2_ photoelectrochemical reduction process, the LSV measurement was performed for the as-prepared nanocomposites. Figure 8(c) shows the LSV results of the RGO-(10, 30 and 50 wt%) SiO_2_@α-Fe_2_O_3_ nanocomposites for the photoelectrochemical reduction of CO_2_. The evaluation of photoelectrochemical CO_2_ reduction of nanocomposites was acquired through current density vs. potential (J–E) behavior of the electrodes. The current potential is measured from 0 to −1.4 V to study the performance of the electrocatalyst for the RHE. The RGO showed cathodic current onset potential at −0.9 V versus RHE. Although the modified α-Fe_2_O_3_ with SiO_2_ and RGO shows more positive onset potential than the bare nanoparticles, the RGO- (10 wt%) SiO_2_@ α-Fe_2_O_3_ exhibited a cathodic onset potential at −0.70 V versus RHE and shifted to −0.58 V versus RHE for RGO- (30 wt%) SiO_2_@ α-Fe_2_O_3_ with respect to the concentration of SiO_2_@α-Fe_2_O_3_ on the RGO. This type of positive shift of onset potential was observed for most of the carbon-based materials and metal catalysts^61^. The high current density of 0.35 mA/cm^2^ was observed for the RGO- (30 wt %) SiO_2_@ α-Fe_2_O_3_ nanocomposites due to the intrinsic effect of the CO_2_ reduction. However, the RGO- (50 wt%) SiO_2_@ α-Fe_2_O_3_ shows less current density due to the agglomeration of the core-shell on the surface of the RGO. The shift of onset potential towards positive is mainly ascribed to the higher evolution of CO_2_ from the saturated solutions as confirmed by a previous report^62^. The effective photoexcited electron transfer from SiO_2_@α-Fe_2_O_3_ through RGO promotes effective photoelectrochemical CO_2_ reduction. Below −0.7 V potential, the RGO and SiO_2_@α-Fe_2_O_3_ nanocomposites cannot achieve a high degree of CO_2_ reduction and there are also no active sites for the formation of hydrocarbon and oxygenates. These results prove that the CO_2_ reduction was carried out by surface oxidation, and there is a significant increase in CO_2_ reduction after the SiO_2_@α-Fe_2_O_3_ nanoparticles were embedded on the RGO sheets. The core-shell formation of α-Fe_2_O_3_ with SiO_2_ enhances the adsorption of CO_2_ species and leads to the effective reduction process. Moreover, the SiO_2_ nanoparticles suppressed the electron recombination of photogenerated electron-hole pairs of α-Fe_2_O_3_, leading to effective enhanced charge transport, which resulted in an enhancement of the photoelectrochemical activity. This enhanced the photoelectrochemical activity due to the suppression of electron recombination, which was well matched with the observed small arc radius with decreased impedance values in the EIS Nyquist plot. Further, the highly conductive RGO favors the effective transfer of photoexcited electrons from the SiO_2_@α-Fe_2_O_3_ nanocomposites by the CO_2_ adsorbed species. The observed potential between 0.6 to −0.7 V indicates the formation of ethanol products as the main product apart from the other products such as CO and HCOO- as reported by Lv. K *et al*.^62^. According to previous reports, the ethanol selectivity leads to a higher degree of surface-bound CO* or CHO*, which is a key step in the formation of C_2_ products. The CHO* generated OC-COOH from HCO_3-_ and further, it produced ethanol by successive electrons from photoexcitation. S7 shows the LSV of the RGO- (30 wt %) SiO_2_@ α-Fe_2_O_3_ nanocomposites in dark and light conditions. This indicates that the shift of onset potential to the positive region was observed with high current density under light irradiation. We found that the light irradiation assists in improving catalytic activity.

### Mechanistic investigation

**Figure 9:**
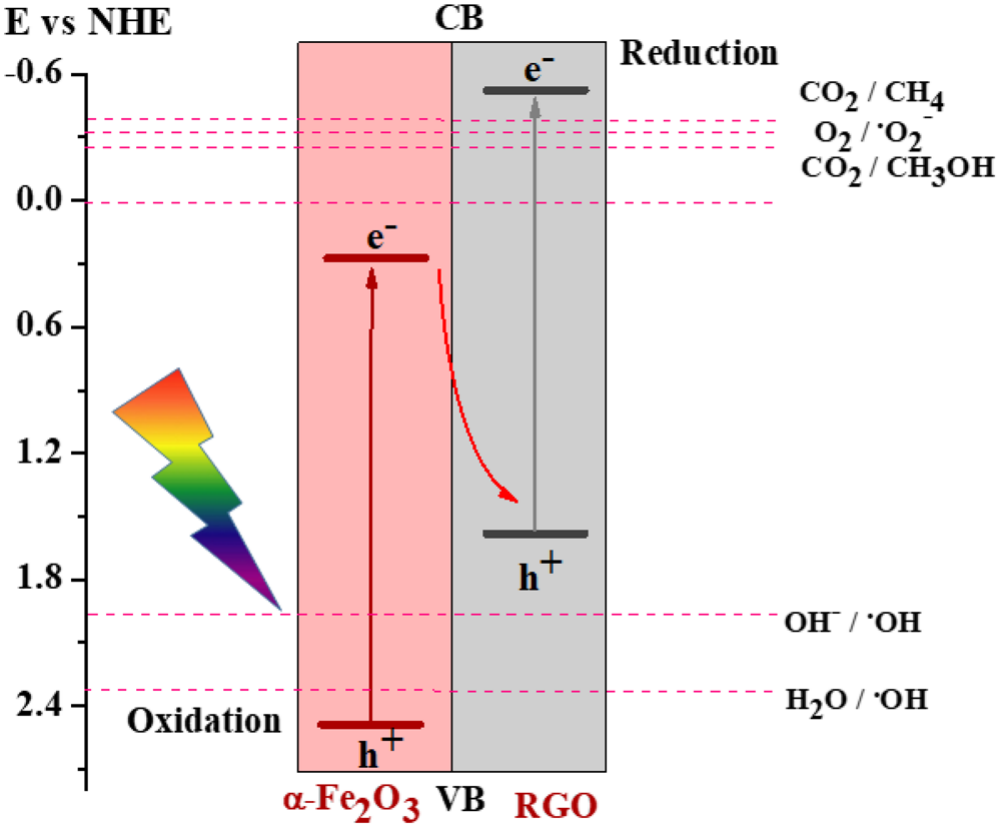
The Z-scheme system of CO_2_ reduction and photo-oxidation using RGO-SiO_2_@Fe_2_O_3_ core-shell nanocomposites.

In contrast to the traditional photocatalytic mechanism, here the mechanism involved in both reduction and oxidation reaction process are interesting due to the formation of heterojuntion. The proposed Z-scheme system for photocatalytic oxidation and reduction reaction of RGO-SiO_2_@α-Fe_2_O_3_ composites is shown in Fig. 9. When irradiating Uv-visible light on RGO-SiO_2_@α-Fe_2_O_3_ composites, the excited electrons could transfer from the CB of RGO to the CB of α-Fe_2_O_3_ and the photoinduced holes would tend to transfer from the VB of α-Fe_2_O_3_ to the VB of RGO. However, CO_2_ cannot be combined with the e− from the CB of α-Fe_2_O_3_ for the reduction process since the CB position of α-Fe_2_O_3_ (+0.31 eV) is more positive than the redox potential of CO_2_/ CH_3_OH (−0.38 eV). Thus, the typical heterojunction mechanism is not suitable for this system. Therefore, we propose the Z-scheme photocatalytic system as shown in Fig. 9. This implies the effective role of the Z-scheme photocatalyst in the reduction and oxidation mechanistic systems. Here, reduced GO can act as a semiconductor that depends on the degree of the reduction of GO (RGO). With the Z-scheme mechanism, the excited electrons in the CB of α-Fe_2_O_3_ transfer to the interface, which combined with the photoinduced holes in the VB of RGO, thus maintaining the strong reducibility of electrons in the CB of RGO and the strong oxidizability of holes in the VB of α-Fe_2_O_3_. Consequently, the efficiency of separation and transfer of electron–hole pairs are enhanced, and the recombination rates of photoexcited electron–hole pairs in both RGO and α-Fe_2_O_3_ themselves are suppressed, which is consistent with the PL and EIS measurement results. Such a Z-shaped electron transfer pathway can significantly improve the photocatalytic activity of the CO_2_ reduction and oxidation process.

The Raman spectra and SEM picture of the RGO-SiO_2_@α-Fe_2_O_3_ composites after the 3^rd^ cycle of CO_2_ reduction as seen in S8 (a) and (b). In the Raman spectra, the G and D phases are stable after the third cycle; however a little reduction in the peak intensity has been observed. The FE-SEM image confirms that there is not much difference observed in the surface morphology of RGO-SiO_2_@α-Fe_2_O_3_ composites after the third cycle of CO_2_ reduction as seen in Fig. S8(b). The recycling results of the RGO-SiO_2_@α-Fe_2_O_3_ (30 wt%) catalyst are presented in S8 (c), and after three cycles little degradation was observed in stability studies.

## Conclusions

SiO_2_@α-Fe_2_O_3_ core-shell decorated RGO nanocomposites with various weight percentages were successfully prepared by the facile sol-gel method and were well characterized. In the photocatalytic activity, the *in-situ* DRIFT setup with thermal effect was utilized to enhance the gas phase reduction of CO_2_. The observed results indicate that more CO_2_ was effectively converted into ethoxide using SiO_2_@α-Fe_2_O_3_ core-shell decorated RGO. Moreover, the nanocomposites also display effective photooxidation of ethanol. The decoration of core-shell nanoparticles on the RGO nanosheets facilitated the formation of heterojunction and effective charge separation of the photogenerated electron-hole pairs. The prepared nanocomposites were applied to photoelectrochemical CO_2_ reduction and photocatalytic gas-phase reduction and oxidation reactions. The photoelectrochemical performance for the reduction of CO_2_ was enhanced after deposition of the core-shell on the RGO nanosheets. The RGO-SiO_2_@α-Fe_2_O_3_ (30 wt%) nanocomposites showed better activity and improved stability for both photoelectrochemical and photocatalytic applications than the other weight percentages of the SiO_2_@α-Fe_2_O_3_ nanocomposites. This fabrication of SiO_2_@α-Fe_2_O_3_ (30 wt%) nanocomposites on the RGO improved the charge carrier’s photothermal harvesting ability, and hence this nanostructure will be a good candidate for photocatalytic reduction and oxidation catalyst systems.

## Supplementary information

In-situ DRIFT investigation of photocatalytic reduction and oxidation properties of SiO2@α-Fe2O3 core-shell decorated RGO nanocomposite.
