# Supplementary material for: In-situ DRIFT investigation of photocatalytic reduction and oxidation properties of SiO2@α-Fe2O3 core-shell decorated RGO nanocomposite
Source: Sci Rep. 2020 Feb 7;10:2128. doi: 10.1038/s41598-020-59037-9 (PMC7005791; doi:10.1038/s41598-020-59037-9)

In-situ DRIFT investigation of photocatalytic reduction and oxidation properties of SiO_2@α-Fe_2O_3 core-shell decorated RGO nanocomposite

Kasimayan Uma[1], Nadarajan Arjun [2], Singaravelu ChandraMohan[3], Guan-Ting Pan[2], Kandasamy Jothivenkatachalam [3], Thomas C.-K. Yang [2*], Ja-Hon-Lin[1*]

[1]Department of Electro-Optical Engineering Engineering, National Taipei University of Technology, Taipei, Taiwan 106

[2]Department of Chemical Engineering and Biotechnology, National Taipei University of Technology, Taipei, Taiwan 106

[3]Department of Chemistry, Anna University, BIT campus, Tiruchirappalli -620024

Corresponding authors: E-mail: ckyang@mail.ntut.edu.tw, Telephone: +886-2-2771-2171 Ext. 2533
jhlin@ntut.edu.tw, Telephone: +886-2-2771-2171 Ext. 4640

S1. XRD of (a) RGO and (b) SiO_2 and (c) α-Fe_2O_3 and (d) SiO_2@α-Fe_2O_3 core-shell nanocomposites. (e) Raman spctra of GO and RGO. (f), (g) and (h) TEM image of SiO_2 , SiO_2@α-Fe_2O_3 and magnificationimage of single core-shell nanocomposites.

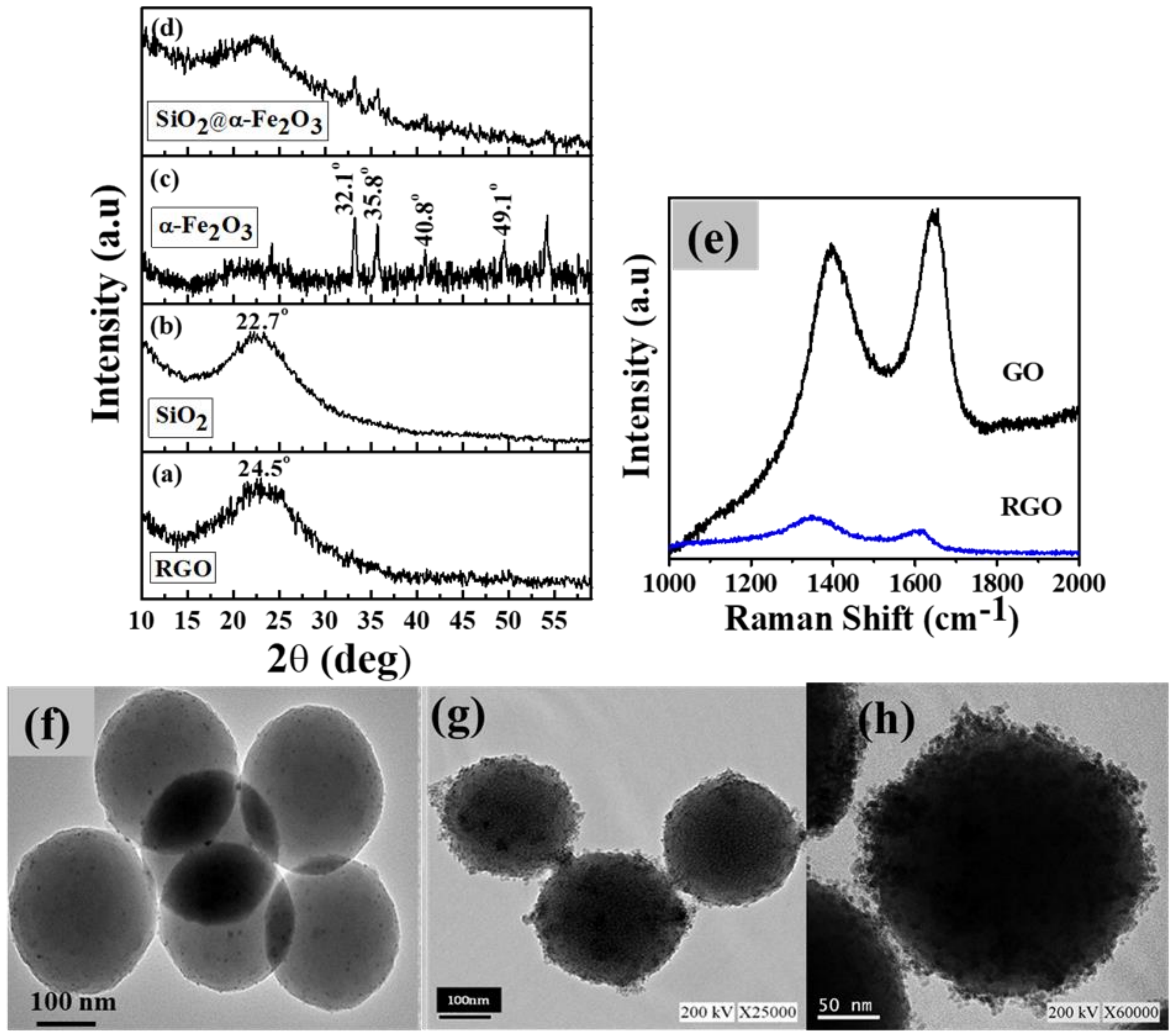

S2. Tauc's plot of RGO, α-Fe_2O_3, RGO-SiO_2@ α-Fe_2O_3 and SiO_2.

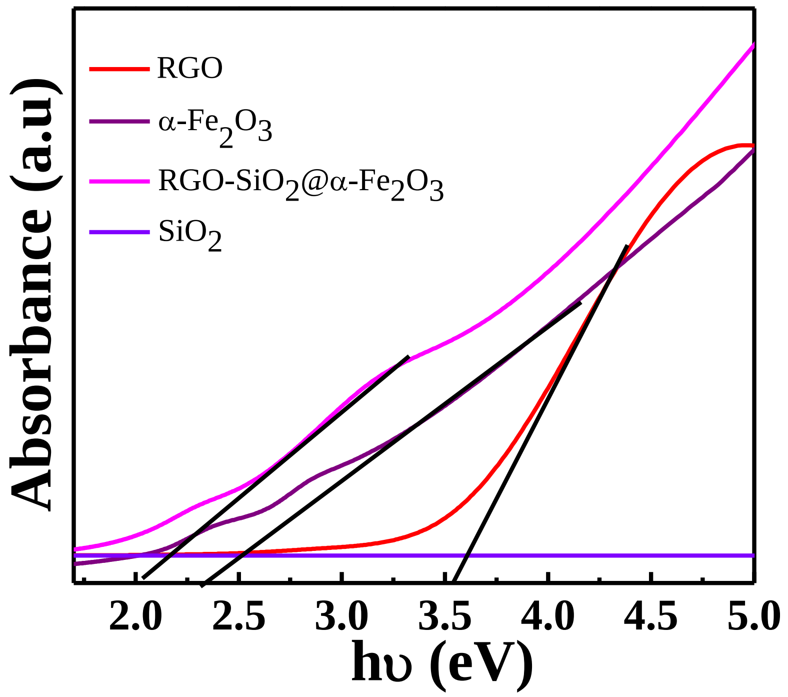

S3. XPS spectra of C1S of (a) RGO, (b) RGO- SiO_2@α-Fe_2O_3, O1s of (c) RGO, (d) α-Fe_2O_3 (e) RGO- SiO_2@α-Fe_2O_3 and (f) Fe2P (f) and (g) Si2p of α-Fe_2O_3, SiO_2@α-Fe_2O_3 and RGO- SiO_2@α-Fe_2O_3.

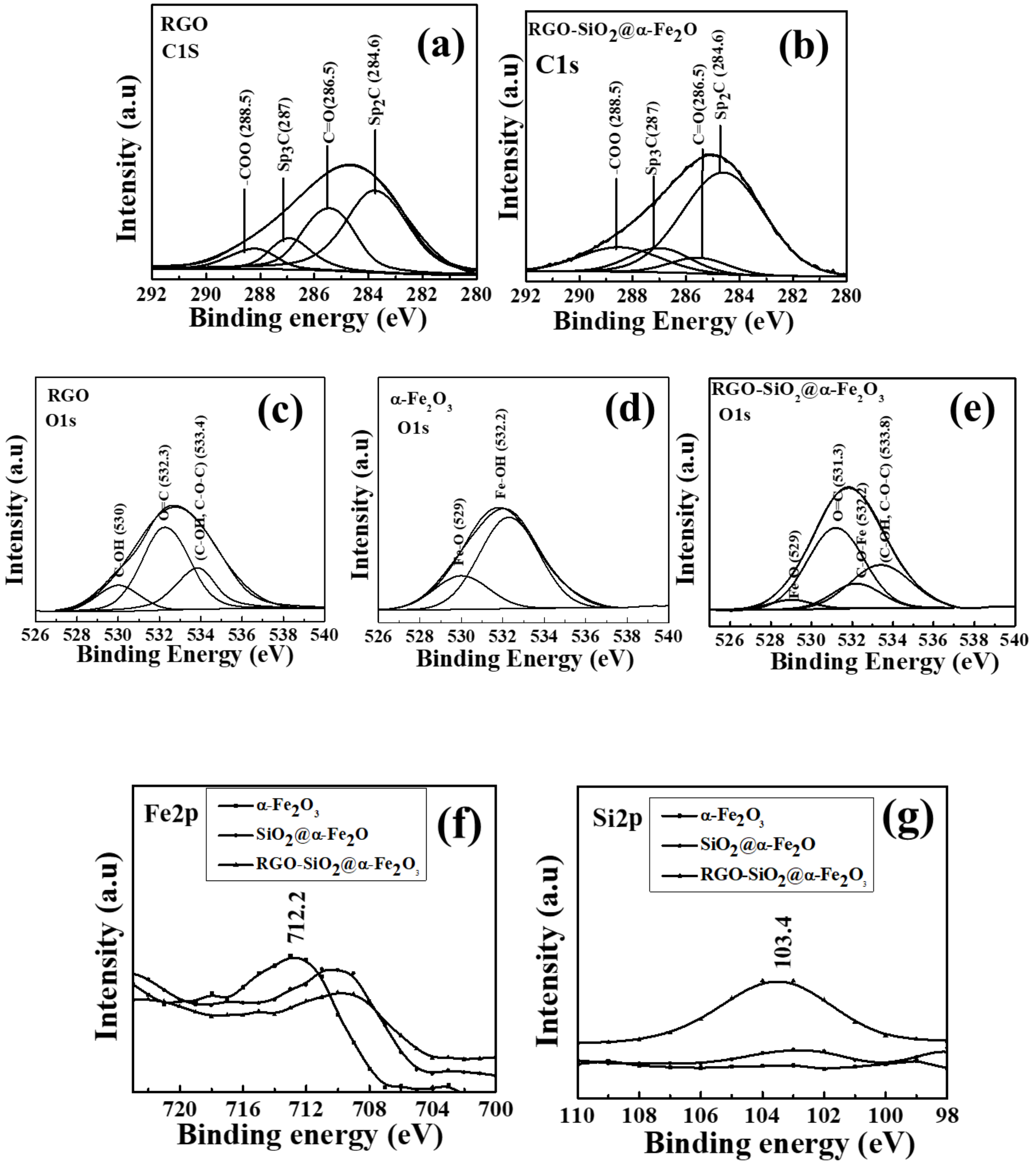

S4. (a) CO_2 reduciton on RGO-(30 wt %) SiO_2@α-Fe_2O_3 core-shell nanocomposites under dark and light condition at 250ºC.

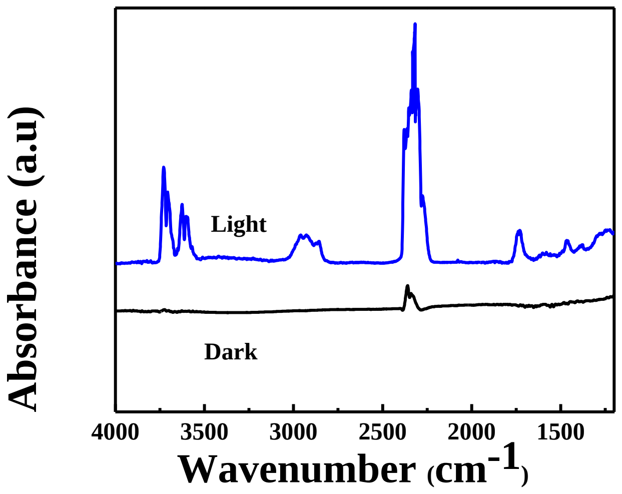

S5. DRIFT-IR spectra of CO_2 reducition on α-Fe_2O_3, SiO_2@α-Fe_2O_3 and RGO-(10, 30 and 50 wt %) SiO_2@α-Fe_2O_3 nanocomposites.

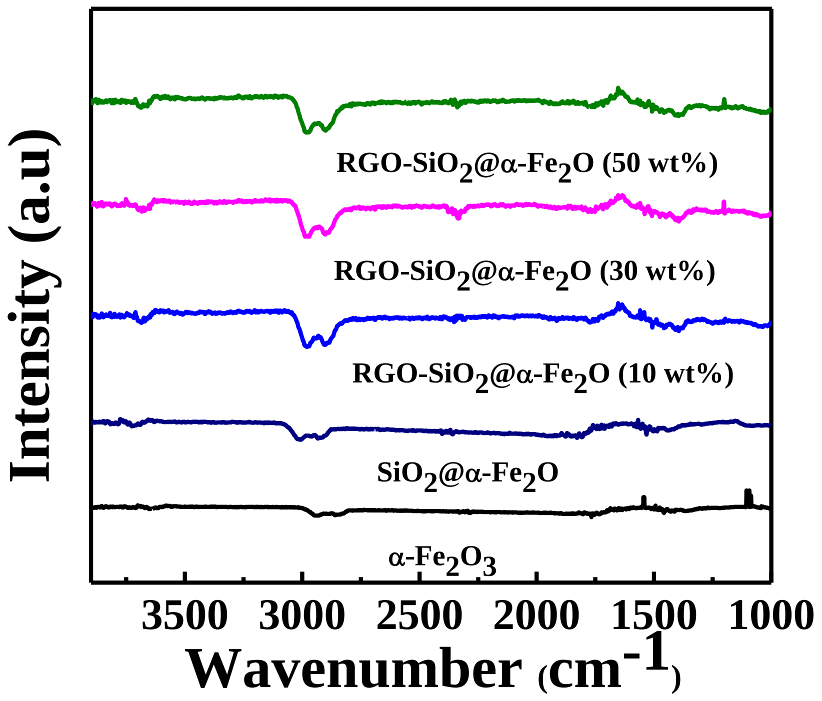

S6. Oxidation of ethanol on α-Fe_2O_3, SiO_2@α-Fe_2O_3 and RGO-(10, 30 and 50 wt %) SiO_2@α-Fe_2O_3 nanocomposites.

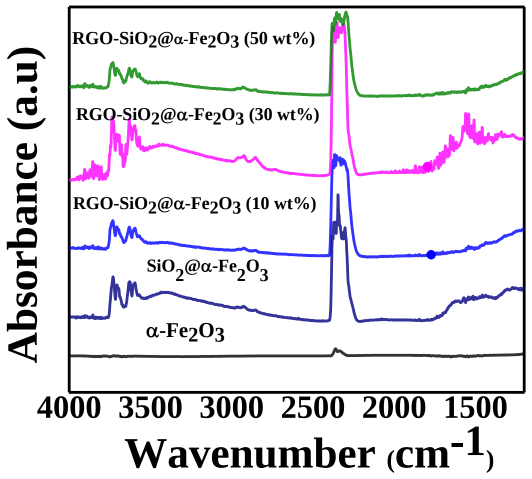

S7. LSV curve of RGO-(30 wt %) SiO_2@Fe_2O_3 nanocomposites under dark and light.

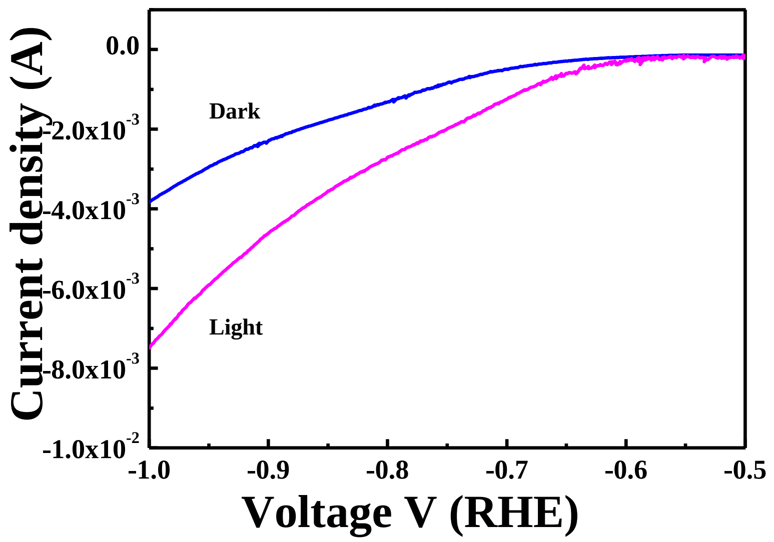

S8. Raman and SEM image of RGO- SiO_2@α-Fe_2O_3 (30 wt%) core-shell nanocomposites after 3rd cycle. The cycles test for the RGO- SiO_2@α-Fe_2O_3 (30 wt%) nanocomposities.

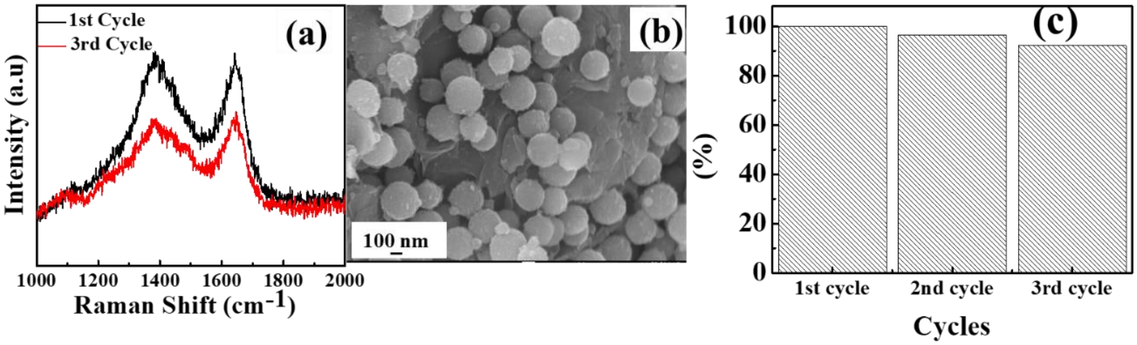

S9. PL spectra of α-Fe_2O_3, SiO_2@α-Fe_2O_3, and RGO- SiO_2@α-Fe_2O_3 (30 wt%) core-shell nanocomposites.

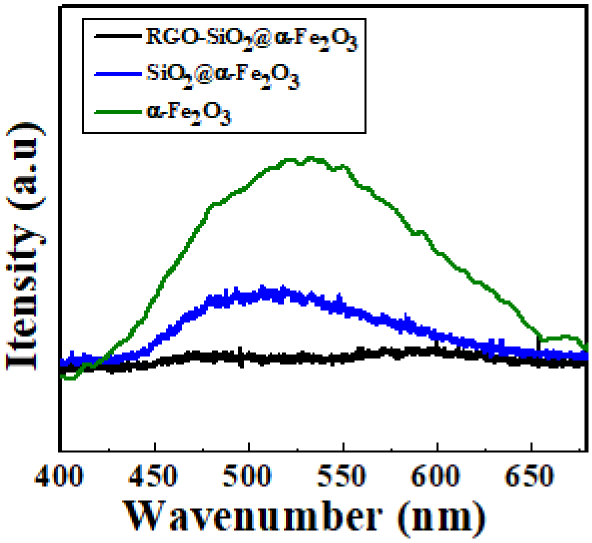

Supplement: Supplementary file 1 — In-situ DRIFT investigation of photocatalytic reduction and oxidation properties of SiO2@α-Fe2O3 core-shell decorated RGO nanocomposite. [file 41598_2020_59037_MOESM1_ESM.pdf]
